# Recent Progress in Mitochondria-Targeted Drug and Drug-Free Agents for Cancer Therapy

**DOI:** 10.3390/cancers12010004

**Published:** 2019-12-18

**Authors:** M.T. Jeena, Sangpil Kim, Seongeon Jin, Ja-Hyoung Ryu

**Affiliations:** Department of Chemistry, Ulsan National Institute of Science and Technology, Ulsan 44919, Koreasangfil@unist.ac.kr (S.K.); sungeon3248@unist.ac.kr (S.J.)

**Keywords:** mitochondria, cancer, drug conjugates, drug-free approach

## Abstract

The mitochondrion is a dynamic eukaryotic organelle that controls lethal and vital functions of the cell. Being a critical center of metabolic activities and involved in many diseases, mitochondria have been attracting attention as a potential target for therapeutics, especially for cancer treatment. Structural and functional differences between healthy and cancerous mitochondria, such as membrane potential, respiratory rate, energy production pathway, and gene mutations, could be employed for the design of selective targeting systems for cancer mitochondria. A number of mitochondria-targeting compounds, including mitochondria-directed conventional drugs, mitochondrial proteins/metabolism-inhibiting agents, and mitochondria-targeted photosensitizers, have been discussed. Recently, certain drug-free approaches have been introduced as an alternative to induce selective cancer mitochondria dysfunction, such as intramitochondrial aggregation, self-assembly, and biomineralization. In this review, we discuss the recent progress in mitochondria-targeted cancer therapy from the conventional approach of drug/cytotoxic agent conjugates to advanced drug-free approaches.

## 1. Introduction

The mitochondrion is probably the most studied eukaryotic cellular subcompartment due to its indispensable role in the regulation of cellular metabolism and multifaceted functions associated with various diseases, such as cancer, neurodegenerative diseases, and diabetes [1,2,3]. Even though each cellular compartment is significant, the mitochondrion is an all-rounder carrying multiple responsibilities including apoptosis (intrinsic) regulation, energy production, respiratory cycle, amino acid metabolism, and redox signaling [4,5,6]. On the one hand, mitochondrion produces ATP (adenosine triphosphate) for cell survival, while, on the other hand, controlling lethal functions, such as apoptosis and necrosis [7,8]. Lethal functions are mainly regulated by a process called mitochondrial outer membrane permeation (MOMP) [9], which occurs during an apoptotic trigger, mediated by the pore-forming activity of proapoptotic proteins, such as the Bcl-2 (B-Cell lymphoma 2) proteins, Bax (Bcl-2 associated X protein), and Bak (Bcl-2 antagonist/killer protein). Mitochondria are critically involved in the initiation of necrotic cell death as well as in events such as abnormal production of reactive oxygen species (ROS), mitochondrial swelling, ATP depletion, and failure of Ca^2+^ homeostasis [10,11]. Mitochondria are key regulators of Ca^2+^ signaling by taking up and releasing Ca^2+^ ions. The increase in the concentration of Ca^2+^ ions in the mitochondrial matrix activates mitochondrial dehydrogenase to stimulate oxidative phosphorylation, and Ca^2+^ ion elevation in the intermembrane space stimulates the uptake of substrates for oxidative phosphorylation [12,13]. Since mitochondria are critical for cellular survival and cell cycle maintenance, they have attracted much attention from biologists and chemists for the past several years.

### Mitochondria-Targeted Cancer Therapeutics

In healthy cells, mitochondria execute a controlled regulation of multiple functions to maintain the cellular growth–death cycle. However, in the case of tumor cells, to meet the higher metabolic demand of rapidly proliferating cells, dysregulation of mitochondrial metabolism occurs [14,15]. The difference between cancer cell mitochondria and normal cells includes several functional alterations, such as mutation of mtDNA that lead to OXPHOS (oxidative phosphorylation) inhibition and thus deficient respiration and ATP generation, mutation of mtDNA-encoded mitochondrial enzymes, such as SDH (succinate dehydrogenase), IDH1 (isocitrate dehydrogenase 1), IDH2 (isocitrate dehydrogenase 2) [16], and structural differences, such as higher membrane potential of cancer cell mitochondria and higher basicity inside the mitochondrial lumen. The evasion of cell death or inhibition of mitochondria-mediated apoptosis is a hallmark for cancer. Mitochondria generate ROS, which is necessary for signaling under normal conditions. However, when apoptosis is inhibited in the case of cancer, ROS contributes to the neoplastic transformation. Furthermore, for supporting tumor cell survival under harsh tumorigenic conditions, such as nutrient depletion and hypoxia, mitochondria provide flexibility in several pathways either by up- or downregulation [17]. This altered mitochondrial metabolism of cancer cells compared with that of their normal counterparts is advantageous for the selective targeting of cancer mitochondria in therapeutics, which focuses on the cancer mitochondria specific features [18]. Directing the therapeutic agent to the mitochondria is an efficient way of eliminating cancer cells because the designed drug molecules could act at the central point of the cell, and therefore, engineering mitochondrial-targeted therapeutic agents have gained much interest in cancer therapy.

Anticancer drugs that selectively disrupt cancerous mitochondria could be achieved by designing molecules that act on the malignant mitochondria by, for instance, inhibiting glycolysis, depolarizing the membrane potential, and inhibiting the mitochondrial permeability transition pore [19,20]. However, penetrating the double-layered membrane of mitochondria requires overcoming certain barriers. The mitochondria membrane consists of a porous outer membrane and a protein-rich inner membrane, which are designed for the tight regulation of its metabolism and to prevent foreign substances from crossing the membrane [21]. To traverse the membrane, the molecules are required to overcome the activation energy associated with the removal of associated water molecules. Molecules with delocalized positive charge can lower this activation energy, which allows them to penetrate the membrane and enter the mitochondria efficiently [22]. The hyperpolarized mitochondrial membrane of cancer cells (−220 mV) compared with that of healthy mitochondria (−160 mV) facilitates the fast and selective entry of positively charged molecules specifically to the cancer mitochondria [23]. The most studied example of such cations is the triphenylphosphonium ion (TPP), which is widely used as a mitochondria-targeting vector. TPP possesses a single positive charge delocalized over the three phenyl groups and stabilized by resonance. Not only the charge but also the hydrophobicity associated with this lipophilic cation favor its interaction with the hydrophobic inner mitochondrial membrane and facilitate its penetration into the mitochondria [24]. Driven by the membrane potential, the concentration of the TPP ion in the cytoplasm increases about 5–10 times compared with that in the extracellular space, which results in an accumulation inside the mitochondria of about 100 to 500 times compared to the cytosol, thereby providing a highly effective mitochondria-targeting system [3]. The advantages of TPP-based systems for targeting mitochondria include the stability of TPP in the biological system, the combination of lipophilic and hydrophilic moieties, low chemical reactivity toward cellular components, and simple synthesis and purification.

**Figure 1:**
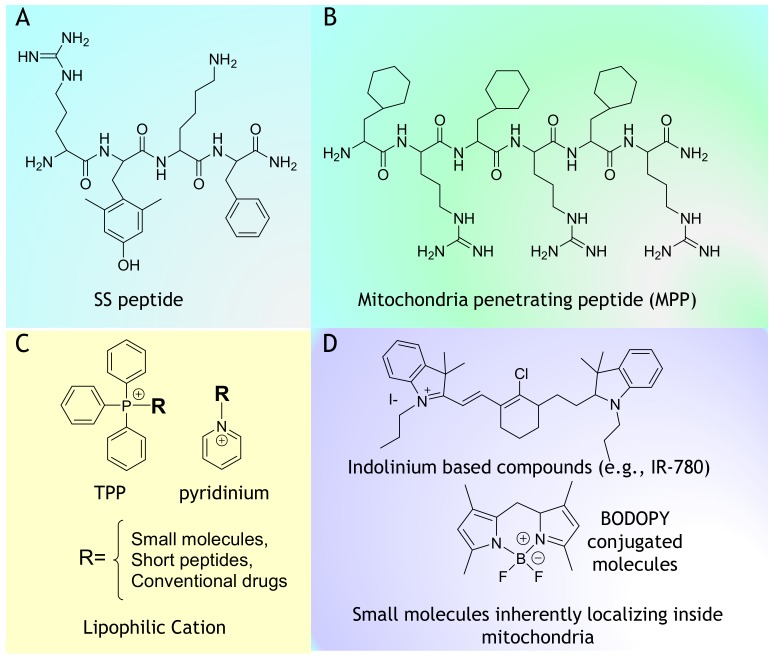
Chemical structure of mitochondria targeting vectors. (**A**) SS peptide, (**B**) Mitochondria penetrating peptide (MPP), (**C**) TPP, pyridinium and Lipophilic Cation and (**D**) Indolinium based compounds (e.g., IR-780) and Small molecules inherently localizing inside mitochondria.

Apart from TPP-based molecules, the rational design of peptides that involves the incorporation of positive and hydrophobic building units provides efficient targeting and mitochondrial accumulation. Szeto–Schiller (SS) peptides were early introduced as mitochondria-penetrating peptides for the delivery of dimethyltyrosine (Dmt) as an antioxidant motif to mitochondria [25,26]. General features of the mitochondria-penetrating peptides (MPPs) include a highly hydrophobic residue such as Fx (cyclohexylalanine) and an alternatively placed positively charged moiety, such as arginine (R) or lysine (K) [27]. The high degree of delocalized positive charge and hydrophobicity enables these peptides to easily cross the plasma membrane to specifically localize inside the mitochondria. Kelley et al. reported that peptides with log P higher than −1.7 could easily localize inside the mitochondria carrying at least a positive charge of +3. Certain small cationic molecules, including rhodamine, pyridinium, and cyanine derivatives, have been shown to exhibit inherent mitochondria-penetrating ability [28,29]. These compounds are extensively used as staining agents, mitochondrial fluorescent probes, imaging agents, and photodynamic therapy (PDT) agents as such or by conjugation with biologically relevant compounds (Figure 1). Furthermore, there are reports describing the nanoscale delivery of anticancer agents to the mitochondria via TPP-modified liposomes, vesicles, or dendrimers [30,31]. Since early introduced strategies for cancer targeting are described in detail in several reviews [1,4,5,17,18], in this review, we focus on recent strategies and progress in targeting mitochondria for cancer therapy. Apart from the conventional approach of inducing mitochondrial damage by a cytotoxic drug or inhibitors, drug-free approaches are introduced as potent mitochondria-targeted anticancer therapeutics. Here, recent mitochondria-targeted therapeutics are classified into two major categories: (1) mitochondria-targeted drug/cytotoxic agents (mito–drug conjugates) and (2) mitochondria-targeted drug-free agents (mito-drug free agents). Motivated by the possibilities of mitochondria as a target for cancer therapy, in this review, we discuss in detail the emerging trend in this field.

## 2. Mito–Drug/Toxic Agent Conjugates for Cancer Therapy

**Figure 2:**
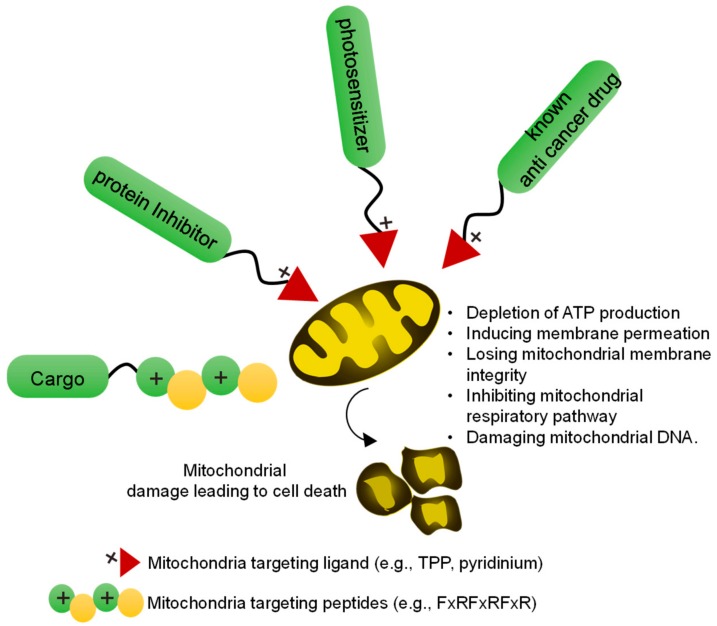
Overview of a mitochondria-targeted drug (Mito–drug)/toxic agent for cancer therapy. In a conventional approach, mitochondria-targeted drugs can be developed by conjugating mitochondria targeting ligands or peptide with the toxic agents. The mitochondria-targeted toxic agents induce mitochondrial damage by different mechanisms leading to cell death.

Mitochondria-targeted anticancer agents were developed by conjugating mitochondria-directing moieties, such as TPP, pyridinium, or cationic peptides, with existing anticancer drugs (doxorubicin, camptothecin, chlorambucil, and cisplatin), mitochondrial protein/metabolism inhibitors, or other cytotoxic agents (Figure 2). In addition, mitochondria-targeted photosensitizers have shown efficient induction of cell death upon light irradiation compared with cytoplasm- or lysosome-targeted analogs, and have, therefore, attracted much attention. Thus, several photosensitizers, such as metal complexes (Ir or Ru), IR 780, BODAPY, and indocyanine dyes, have been directed to the mitochondria and tested for tumor curing, showing improved results compared with analogs targeted to the cytoplasm or other organelles, especially under hypoxic condition.

### 2.1. Conventional Drugs Targeting Mitochondria

**Figure 3:**
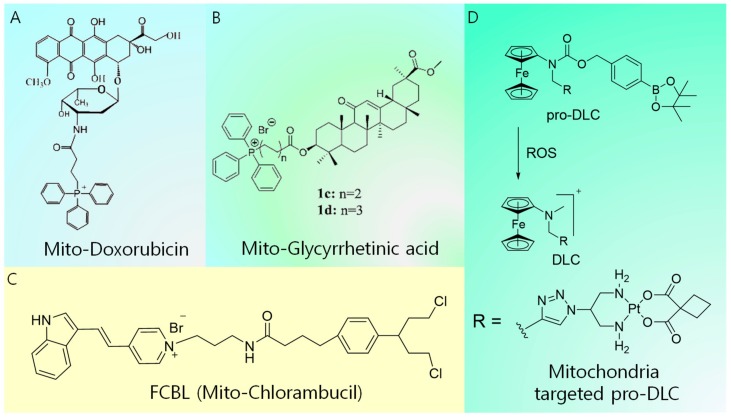
Summary of molecular design of mitochondria-targeted conventional drugs. (**A**) Mitochondria-targeted doxorubicin, (**B**) mitochondria-targeted glycyrrhetinic acid, (**C**) mitochondria-targeted chlorambucil, and (**D**) mitochondria-targeted pro-DLC.

Conventional chemotherapy drugs, including doxorubicin (DOX), Pt-based derivatives, camptothecin, and chlorambucil, suffer from dosage limit, development of resistance, and undesirable side effects. Their delivery to mitochondria is of pivotal importance to improve their efficacy via different mechanisms, such as the depletion of ATP production, induction of membrane permeation, loss of mitochondrial membrane integrity, inhibition of the mitochondrial respiratory pathway, and damage of mitochondrial DNA case by case. Lavasanifar et al. developed TPP-conjugated DOX to overcome drug resistance (Figure 3A) [32]. The conjugation of TPP with DOX enhanced the intracellular accumulation capacity of the latter and afforded enhanced cytotoxicity toward both DOX-sensitive cells and DOX-resistant cells. The TPP–DOX system was shown to preferentially accumulate in the mitochondria of both DOX-sensitive and DOX-resistant cells. However, free DOX showed higher cellular uptake in DOX-sensitive cells and accumulated in the nucleus. A cytotoxicity analysis in MDA-MB-435 showed that TPP–DOX exceeded the cytotoxicity of free DOX after 72 h of incubation. Furthermore, the resistance index (ratio of IC_50_ of the sample of interest to the IC_50_ of free DOX) of TPP–DOX in MDA-MB-435/DOX underwent a noticeable reduction (67.7 after 72 h) compared with that of free DOX (152 after 72 h). Meanwhile, an anticancer drug based on glycyrrhetinic acid exhibits an inhibitory effect on cancer cell lines by inducing autophagy and activating the interaction between CD 95 and CD 178, thereby leading to apoptosis. However, the use of glycyrrhetinic acid is challenging because of its poor solubility and limited cellular accumulation. These issues were solved by Wang et al. through the design of a mitochondria-targeted analog of glycyrrhetinic acid (Figure 3B) [33]. They developed a series of new glycyrrhetinic acid derivatives that conjugate with TPP to specifically target the mitochondria of tumor cells. These compounds exhibited better accumulation and selectivity toward cancer cells. Moreover, they significantly induced cell cycle arrest at the G2/M phase, leading to the inhibition of cancer cell proliferation [33]. The mitochondria-damaging drug F16 ((E)-4-(1H-Indol-3-ylvinyl)-N-methylpyridinium iodide) and DNA-damaging agent chlorambucil (CBL) were covalently linked, resulting in the mitochondria-targeting anticancer agent Mito-Chlorambucil (FCBL) (F 16 conjugated with CBL resulting in FCBL or mitochondria targeted CBL) (Figure 3C) [34]. FCBL accumulates in the mitochondrial matrix, damaging mtDNA, and consequently inducing apoptosis. Recently, Mokhir et al. introduced a concept called pro-delocalized charge (pro-DLC), which involves the activation of delocalized positive charge within the cell to generate delocalized charge (DLC) (Figure 3D). This concept is based on the *N*-alkylamino ferrocene structure, in which the prodrugs are activated by the reaction with ROS within the cell to produce DLC [35]. Since ROS are overexpressed inside the cancer cells, higher cancer selectivity could be achieved. A conjugate of a pro-DLC agent with the clinically approved drug carboplatin was synthesized, and its accumulation inside the cancer cell was confirmed to be higher than that of the free drug. Moreover, a fluorescence analog of pro-DLC accumulated inside cancer mitochondria with a Pearson coefficient of +0.8. The anticancer activity of pro-DLCs was substantially higher than that of the parent carboplatin. Furthermore, the pro-DLC agents exhibited a 2.7-fold increase in transmembrane potential (∆Ψ_c_) of A2780 cells and reduced intracellular glutathione (GSH). Generally, positively charged mitochondria-targeted ligands work fine under in vitro conditions. In contrast, during in vivo administration, the positive charge causes nonspecific serum protein interactions, reducing the tumor-targeting efficiency. In this regard, the pro-DLC approach offers better selectivity for targeting cancer as the positive charge is activated only at the site of the tumor.

### 2.2. Molecules Targeting Mitochondrial Metabolism, Functions, or Proteins

Mitochondrial metabolic functions are critical for malignant cell growth; hence, targeting the metabolic pathway is a potent strategy to inhibit cancer growth. Numerous proteins necessary for the metabolism and energy production of mitochondria are present in their inner/outer membrane. The alterations of these metabolic factors affect malignant cell functions, thereby leading to cell death. A few methods have been developed to target mitochondrial respiration or other relevant functions that can disrupt the cancer cell mitochondria.

It is known that mitochondria are the power source of eukaryotic cells, using the oxidation of glucose to produce ATP. ATP is generated by the transfer of electrons from nicotinamide adenine dinucleotide (NADH) to oxygen by a series of large proteins, which occurs in the mitochondrial inner membrane and is catalyzed by ATP synthase. Since the electron respiratory chain is related to the capture of electrons by oxygen, it is also associated with the production of reactive singlet oxygen. Thus, compounds that can interfere with the respiratory chain would induce oxidative stress, leading to dysfunction of mitochondria. In this context, alpha-tocopheryl succinate (α-TOS), a vitamin E analog that binds to the ubiquinine binding site of complex III and inhibits succinate dehydrogenase (SDH), is used as a drug to induce the accumulation of ROS [36]. Dong et al. reported that vitamin E analogs selectively inhibit the proliferation of diverse malignant cells [37]. In addition, α-TOS inhibits Bcl-Xl and Bcl-2 by blocking the BH3 domain. Since higher esterase activities in normal cells compared with the cancer cell can hydrolyze α-TOS to α-TOC (alpha-tocopherol), α-TOS exhibits reduced toxicity toward normal cells. As another inhibitor, resveratrol inhibits complex III and decreases ROS levels. Resveratrol is a natural antioxidant obtained from berries, and it is known to reduce oxidative stress by inducing the expression of mitochondrial superoxide dismutase (SOD2) in mitochondria [38]. Although the effect of ROS is still undefined, resveratrol induces apoptosis through the mitochondrial pathway, which is associated with tumor inhibition.

The maintenance of the mitochondrial membrane potential is regulated by the opening and closing of the ion channel, mitochondrial permeability transition pore (MPTP) [39]. The abnormal opening of MPTP induces the collapse of the mitochondrial membrane potential and release of cytochrome c, an apoptotic factor, into the cytosol. Cancer cells have specific MPTP characteristics, which are different from those of normal cells. One of them is the decrease in the function of the adenine nucleotide transporter (ANT), which plays an important role in adenine exchange. Some studies have been conducted to induce the apoptosis of cancer cells by targeting these specific traits of MPTP [40]. Lehenkari et al. reported that clodronate, a bisphosphonate analog, can inhibit the activity of ANT and the mitochondrial oxygen consumption, and depolarizes the mitochondrial membrane potential [41]. The intracellular metabolism can transform clodronate into a β-γ-methylene analog of ATP, which is a cytotoxic agent for macrophages. Although the molecular basis for the cytotoxic effects of the clodronate metabolite AppCCl_2_P has not been determined yet, these authors found that AppCCl_2_P inhibits adenosine triphosphate (ADP)/ATP translocation, causing apoptosis upon long treatment. Meanwhile, Bcl proteins, which are located in the mitochondrial inner membrane, regulate the programmed cellular death. They are overexpressed in many cancer cells, contributing to tumor initiation progression and resistance to cancer therapy [42]. For example, expression of the Bcl-X_L_ protein is associated with drug resistance of cancer cells, and the reduction of Bcl-2 increases the sensitivity to anticancer drugs. ABT-737, a small inhibitor of the antiapoptotic proteins Bcl-2, Bcl-X_L_, and Bcl-W, was discovered to enhance the effects of cytotoxicity in synergy with chemo- and radiotherapy [43]. In addition, ABT-737 was shown to disrupt the mitochondrial membrane potential, diminish intracellular GSH, and increase ROS production.

Targeting overexpressed mitochondrial proteins in cancer cells is an alternative method to achieve cancer selectivity. Among the hundreds of proteins present in the mitochondria, a group of proteins known as chaperons attracts much attention due to the dependence of cancer cells on the prosurvival functions of these proteins. A mitochondrial analog of HSP90 (a molecular chaperon), TRAP1, is overexpressed in cancer cells, suppresses the cell death, and reorganizes the metabolic pathway. Kang et al. reported that the major chaperon expressed in the cancer cells is TRAP 1, not HSP90. They designed a mitochondria-targeting HSP90 inhibitor called SMTIN-P01 by conjugating TPP, which selectively inactivates mitochondrial TRAP1 [44]. Terpyridine–platinum complexes have been reported to inhibit thioredoxin (TrxR) effectively [45]. In this work, the rational design of TPP conjugation to terpyridine-Pt (TTP) provided the synergistic inhibition of both mitochondria and glycolytic metabolism in cancer. TTP showed enhanced toxicity toward cisplatin-resistant ovarian cancer cells and preferentially inhibited TrxR. The TEM images recorded after treating with TTP showed that the mitochondria of Caov3 cells were severely changed to an onion-like structure with multilamellar folding. The expression of mitofilin (IMMT), a cristae morphology regulator, was increased by TTP. Furthermore, TTP induced autophagy and mitophagy after treatment. The membrane potential of mitochondria was dissipated, and ATP and ROS production was lowered after treatment with TTP. TTP inhibited both mitochondrial and glycolytic bioenergetics of cancer cell metabolism, causing cell death. The mitochondria metabolism or protein targeted drugs, and their target of interests are summarized in Table 1.

### 2.3. Mitochondria-Targeted Peptides

Peptide drugs attract the attention of biomedical researchers due to their ease of synthesis, size and functional group tunability, and biodegradability [46,47,48]. Therefore, mitochondria-targeting agents based on peptides possess remarkable advantages over other mitochondriotropics, which includes biocompatibility and facile synthesis. For a peptide to penetrate the mitochondrial membrane, this peptide should be rationally designed to have optimum positive charge and hydrophobicity [27]. This could be achieved either by incorporating lipophilic cations, such as TPP (limited to short sequence), to the peptide or by including positively charged (argininine) and hydrophobic moieties (cyclohexyl alanine) in the peptide design, which results in a class of peptides known as MPPs [18,27].

Horton et al. developed a class of synthetic peptides that exhibit efficient cellular uptake and specific mitochondrial localization. The latter was studied to provide an important conceptual framework for understanding how mitochondrial localization could be achieved for synthetic compounds. The peptides were designed to have two crucial properties, i.e., cationic charge and lipophilicity. Previous studies of cell-penetrating peptides (e.g., TAT) indicate that the incorporation of arginine boosts the charge-driven cellular uptake by providing cationic charge to cross the plasma membrane, which exhibits a potential gradient that can transport cationic species by electrophoresis from the extracellular space into the cell. The mitochondrial membrane possesses similar characteristics with a negative potential gradient across the membrane, which facilitates the passing of the cationic moieties through the membrane. However, the inner membrane of mitochondria is more hydrophobic than the plasma membrane, which restricts the entry of most cell penetrating peptides (CPP) or cationic moieties into the mitochondria. Thus, the molecules should be rationally designed to exhibit enough hydrophobicity. Horton et al. designed a series of peptides by incorporating lysine (K) and arginine (R) for positive charge and phenylalanine (F) and cyclohexylalanine (Fx) for hydrophobicity. Careful analysis revealed that compounds possessing a cationic charge of +3 or above and a log P value of approximately −2 localized inside mitochondria, whereas below those values, compounds were excluded from the mitochondria, instead appearing in the lysosome or nuclei [27].

**Figure 4:**
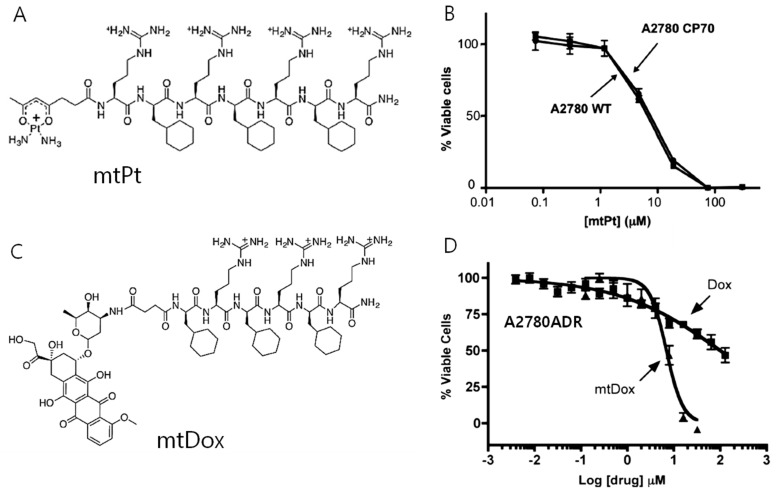
(**A**) Chemical structure of mitochondria-penetrating peptides (MPPs) conjugated with anticancer drug cisplatin (mtPt), (**B**) cell cytotoxicity of mtPt in A2780 WT(wild type) and cisplatin-resistant A2780CP70 cell for an incubation time of 72 h showing similar cytotoxicity in both cell lines, (**C**) chemical structure of MPP conjugated with anticancer drug doxorubicin (mtDox), (**D**) cell cytotoxicity of mtDox in A2780ADR for a treatment period of 24 h showing higher cytotoxicity of mtDox compared with Dox. (Copyright permission received from reference [51] and [52]).

MPPs can be utilized upon conjugation with a variety of cargo molecules as the transport to mitochondria. However, the covalent conjugation of small molecules to MPPs can negatively affect the activity of the appended cargo against the cellular target. Therefore, Kelley’s group reported the conjugation of small molecules with MPPs using cleavable disulfide linkers for small molecule delivery [49]. Chamberlain et al. conjugated DOX with MPPs for inhibition of DNA topoisomerase II inside mitochondria (Figure 4A,B) [50]. This inhibition normally occurs in nuclei for conventional DOX. However, this system exhibited reduced activity in a cell line that expresses a common efflux pump. On the other hand, mitochondria-targeting DOX (mtDOX) provided a means to limit drug efflux and inhibited mitochondrial DNA topoisomerase even in the efflux pump overexpressed cells. The same group reported that mtDOX eliminates nuclear effects associated with cardiotoxicity leading to mitochondrial biogenesis [51]. Although DOX has a high level of activity against cancer cells, side effects, such as dose-limiting cardiotoxicity, are a bottleneck for clinical usage. This is linked to the downstream effects of nuclear DNA damage. These authors demonstrated that mtDOX lacks any direct nuclear effects, and can induce mitochondrial biogenesis, preventing cellular death in cardiomyocytes. Platinum-based anticancer agents are also being developed by Kelley’s group as mitochondria-targeting drugs [52]. Kelly’s group developed an analog of platinum conjugated with MPP for the delivery of the anticancer drug cisplatin (mtPt) into mitochondria of cancer cells (Figure 4C,D). As a result, mtPt induced apoptosis of cancer cells specifically without damaging nuclear DNA and mediated the activity of platinum-based chemotherapy.

### 2.4. Mitochondria-Targeted Photosensitizers

In comparison to the conventional therapeutic strategies for cancer treatment using cytotoxic small molecular drugs, PDT offers high selectivity, no severe side effect, and rapid action [53,54,55]. PDT is a process consisting of two nontoxic components, a photosensitizer (PS) that acts like a drug and a light source of a suitable wavelength, whose combination induces cancer cell death. The PSs are localized inside the cell or subcellular compartment and are nonactive until excited by a photon of a suitable wavelength. Upon the irradiation of light, the excited PSs transfer the energy to molecular oxygen to generate ROS. The oxygen (O_2_) present in the tumor microenvironment is converted into singlet oxygen (^1^O_2_), causing irreversible damage to the tumor tissue [56]. This process occurs only in the particular site where the light is irradiated, and hence, PDT agents cause fewer side effects than conventional drugs. However, there is an intrinsic barrier for the PDT to be operative in the tumor site. The tumor microenvironment is generally hypoxic in nature, which hampers the efficient production of toxic singlet oxygen. Inhibition of the mitochondrial respiratory pathway has been found to increase the production of intramitochondrial oxygen, which in turn enhances the efficiency of PDT. Thus, mitochondria-targeted PDT has attracted much attention compared with other cellular targets or subcellular targets [57]. PDT agents can be modified with metal complexes or porphyrins having lipophilic cations (TPP, pyridinium), cyanine, or IR-780-based PSs. Conjugation of PSs with cationic peptides is the commonly adopted method for directing the PS inside the mitochondria of the cell.

#### 2.4.1. Mitochondria-Targeted Metal Complexes for PDT

Lv et al. compared the efficiency of mitochondria- and lysosome-targeted iridium complexes under hypoxic conditions. Mitochondria-targeted iridium complex Ir-P(Ph)_3_ (an iridium (II) complex conjugated with triphenylphosphonium) exhibited high therapeutic efficiency compared with that of a lysosome-targeted Ir-alkyl complex upon light irradiation. After treatment with Ir-P(Ph)_3_ followed by 15 min of light irradiation (475 nm), 3.0% dead cells were detected, whereas only 0.15% dead cells were detected upon treatment with Ir-alkyl under hypoxic condition. As expected, irradiation under normoxia increased the percentage of dead cells compared with hypoxia conditions. This result suggests the higher therapeutic efficiency of mitochondria-targeted Ir compounds. Under hypoxia conditions of a tumor, where the oxygen concentration in the solid tumor is as low as 4%, the cell-killing ability of PDT agents was much lower than the expected efficiency judging from their quantum yield studies. The possible explanation for the high cell killing ability upon targeting mitochondria is that the mitochondrial respiratory system might be inhibited by the PDT agents, leading to high intramitochondrial oxygen concentration, which favors the singlet oxygen production to induce cell death even under hypoxic conditions [58].

Among the variety of transition metal complexes, Ir-based complexes are normally selected due to their high photostability, large stock shift, and high ^1^O_2_ quantum yield. However, due to their hydrophobic nature, iridium complexes often aggregate inside the hydrophilic biological environment. To address this issue, Hoe et al. designed gemini iridium (III) complexes (GIC) with adjustable water solubility and excellent self-assembling property (vesicle structure with inhibited aggregation property), and applied them for imaging and PDT effect [59]. To provide the Ir complexes with water solubility and inhibit their aggregation-induced quenching, quaternary ammonium groups were conjugated with the ligands (CN and NN) of Ir complexes. The designed GIC showed good mitochondria localization and PDT effect both in vitro and in vivo upon light irradiation. Furthermore, the renal clearance efficiency of GIC Ir complexes was found to improve due to their high water solubility property.

Apart from Ir-based metal complexes, Ru complexes are widely employed for PDT studies [60]. Cationic lipophilic Ru complexes can localize inside the mitochondria. Zhou et al. reported that integration of a chloromethyl group into the [Ru(II)(bpy)_3_]^2+^ complex localized inside the mitochondria produces carbon radicals (type I mechanism) upon light irradiation in the presence of reductants. NADH, abundant in mitochondria, acts as a reductant by photoinduced electron transfer from NADH to the chloromethyl-modified Ru (II) complex. Furthermore, the scarcity of NADH disrupts redox homeostasis and enhances apoptosis [61]. The majority of clinical and preclinical PSs, mainly porphyrin derivatives, generate toxic ROS, singlet oxygen (^1^O_2_), via the type II mechanism (energy transfer between PS and O_2_), which highly depends on the oxygen concentration. However, the type I mechanism includes the production of superoxide anion radicals (O_2^−^_•) and hydroxyl radicals (OH•) generated by electron transfer between the excited PS, O_2_, and biomolecules. Hence, type I mechanism-based PSs deserve more attention to address hypoxia condition. Organo–ruthenium complexes coordinated by polypyridyl ligands produce higher singlet oxygen yield as a result of their highly populated triplet metal-to-ligand charge transfer state. Ruthenium complexes have recently attracted much attention due to their DNA intercalation capacity and unique photophysical properties favorable for PDT. For instance, Weil et al. presented a macromolecular approach by combining the [Ru(bpy)_3_]^2+^ complex on a protein carrier scaffold decorated with a mitochondria-targeting moiety [62]. This approach afforded improved phototoxicity against the acute myeloid leukemia cell line, which is an aggressive disease leading to death in up to 8 out of 10 patients. A bioinspired molecular design was introduced, cHSA-PEO-TPP-Ru, in which the blood serum protein (HAS) acts as an efficient transporter of PS. The complex cHSA-PEO-TPP-Ru showed an IC_50_ of 34.9 ± 2 nM under light irradiation, which is the best value reported. In contrast, the control Ru complex without any modification showed an IC_50_ of 7.7 ± 1.3 μM. Furthermore, cHSA-PEO-TPP-Ru showed a five times higher two-photon absorption cross section, suggesting its applicability for localized photochemical reactions beneath the skin with minimum off-target damage.

#### 2.4.2. Mitochondria-Targeted Small Molecules for PDT

Autofluorescence in the visible light region, lack of deep tissue penetration (near IR region), and poor blood clearance are problems associated with PDT based on heavy metal atoms. In contrast, small molecules, such as cyanine dye or boron-dipyrromethane (BODIPY), can overcome these issues, since they often show good deep tissue penetration and no blood clearance issues [63]. Kim et al. developed a mitochondria-targeting brominated near infrared NIR fluorophore (MitDt) for enhanced PDT treatment, which allows rapid clearance and stays localized in the mitochondria for long periods. The molecular design of MitDt consists of triphenylphosphonium (TPP) conjugation on the heptamethine meso-position of a cyanine dye for enabling mitochondria localization, and a bromide ion is conjugated with the indolenine group, which enhances the production of ROS by the heavy atom effect during light irradiation with a 662 nm laser. MitDt showed no dark toxicity toward cancer cells, but cell viability was reduced to (48.33 ± 7.39)% under light irradiation (662 nm, 100 mW cm^−1^, 5 min). The in vivo applicability of MitDt was evaluated in nude BALB/c mice (NCl-H460 model). Within 1 h of injection, MitDt distributed throughout the body. When time elapsed, MitDt was rapidly cleared from most of the organs except from the tumor, according to a biodistribution analysis. The tumor size of the MitDt-treated group was reduced to 719.41 ± 21.20 mm^3^ compared with that of the control (2499.90 ± 519.77 mm^3^), suggesting the high tumor-suppressing efficiency of MitDt. The MitDt fluorophore, which stays in the tumor for a prolonged time, disrupts the mitochondria, which results in significant tumor reduction due to ROS production during laser irradiation [64].

Although the indocyanine dyes are promising as NIR PDT agents, their applications are limited due to poor water solubility and dark toxicity. Thomas et al. introduced highly water-soluble indocyanine derivatives for PDT with enhanced photostability and mitochondria-targeting ability. The indocyanine dye IR-780 was modified with a pyridinyl group to generate the mitochondria-targeting molecule IR-Pyr. To achieve cancer selectivity, IR-Pyr was, in turn, modified with hyaluronic acid (HA). HA has been extensively used for selective cancer targeting due to the overexpression of HA receptors (CD44) in cancer cells. The electrostatic interaction between positively charged CD44 and negatively charged HA generates micellar aggregates of HA-IR-Pyr. The HA-IR-Pyr aggregates showed an IC_50_ of 5–7 μM in HeLa and MDA-MB 231 cell lines under PDT conditions (808 nm laser, 3 min irradiation, 200 mW), and no activity toward normal cells. In vivo administration of HA-IR-Pyr followed by laser irradiation also showed significant tumor reduction, which renders it a good candidate for mitochondrial-targeted PDT with high water solubility [65]. The same group developed another powerful photodynamic therapeutic agent with a synergistic effect. They designed a conjugate of a heat shock protein inhibitor and a PS, named as IR-PU. Since heat shock proteins are overexpressed in cancer cells, the designed molecule IR-PU showed selective accumulation in cancer cells, possessing an antiapoptotic property. Two relevant heat shock proteins, HSP 90 and TRAP 1, express in both mitochondria and cytoplasm, and therefore, the rationally designed molecule IR-PU provides simultaneous inhibition of both cytoplasmic and mitochondrial proteins. This group of proteins acts as molecular chaperons and facilitates tumor growth and propagation. IR-PU binds with the HSP90 protein family in the cancer cells to inhibit their chaperon activity, while mitochondria-targeted PDT molecules rapidly damage their biological activity, thus offering a synergistic impact. Different mitochondria-targeted conventional drugs and their advantages/disadvantages are summarized in Table 2.

## 3. Mitochondria-Targeted Drug-Free Agents

**Figure 5:**
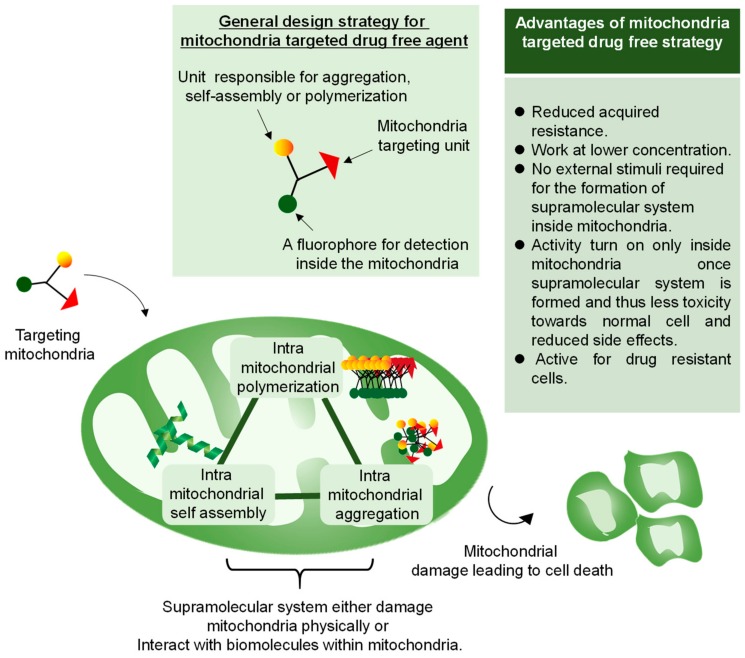
Overview of a mitochondria-targeted drug-free approach. In this approach, the monomer is designed with the ability to either polymerize, self-assemble, or aggregate inside the cancer mitochondria taking advantage of cancer mitochondria characteristics such as higher negative membrane potential, higher basicity inside the matrix or overproduction of ROS. The resulting supramolecular system leads to mitochondria damage, causing cell death.

Clinical treatment of cancer typically involves the use of anticancer cytotoxic drugs that can kill cancer cells by damaging or interfering with DNA. Unfortunately, this treatment system does not afford ideal results, judging from the increasing percentage of deaths caused by cancer every year [66]. A large number of patients do not respond to the currently available therapies and have severe side effects. Molecular-targeted drugs based on ligand–receptor interactions, which were expected to be effective as antitumor drugs, were introduced in the 2000s. In fact, they inhibit molecules or proteins related to cancer cell growth and have fewer side effects than conventional chemotherapy. However, their repeated administration causes alternation of target proteins, leading to the development of acquired drug resistance, with the concomitant drug inactivity [67]. Therefore, it is critical to developing an alternative strategy that could overcome the barrier imposed by conventional drugs for chemotherapy. Research on new therapeutic systems is now focusing on the so-called “drug-free” approach. In this approach, certain in situ macromolecular systems are introduced or formed in situ inside the cell or subcellular compartments to lead to cell death either by physical damage or by inhibition of the metabolic function. Self-assembly, aggregation, polymerization, and biomineralization inside the cancer cells are emerging examples that could tackle cancer cells efficiently [68]. In the drug-free approach, the anticancer activity turns on once the macromolecular system interacts with the targeted cancer cell component while being inactive in their molecular state, thus inducing fewer side effects both in vivo and in vitro. The selective targeting of mitochondria over cytoplasm or other cellular components attracts special attention in the drug-free approach due to properties of mitochondria, such as basic condition, a negative membrane potential that differentiates them from normal cells, and a feasible size of 500–1000 nm that allows a macromolecular system to be formed inside the mitochondria by designing a proper molecular structure. Targeting mitochondria by the drug-free approach provides an efficient way of overcoming drug resistance by decreasing the drug efflux by forming a supramolecular system inside the mitochondria, leading to cell death via different mechanisms including depletion of ATP production, which is primary for cell survival, loss of membrane integrity, and release of cytochrome C and apoptotic proteins. Since most of the mitochondria system does not rely on a specific target protein, the possibility to develop acquired resistance during the clinical administration is lower, thus offering an efficient anticancer strategy. Intramitochondrial aggregation, intramitochondrial self-assembly, and intramitochondrial biomineralization are mitochondria-targeted drug-free strategies, which are discussed below (Figure 5).

### 3.1. Aggregation of Rationally Designed Molecules Inside the Mitochondria

**Figure 6:**
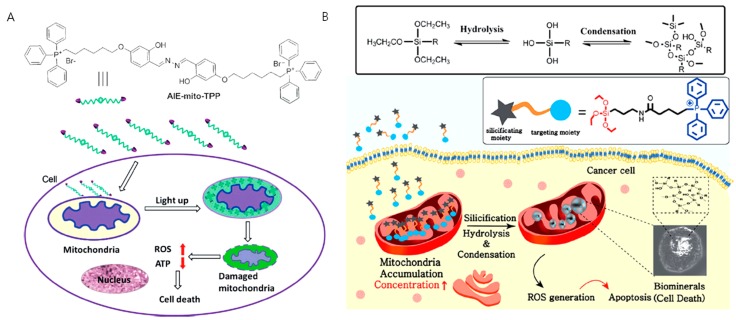
Figure showing (**A**) intramitochondrial aggregation with a rationally designed probe AIE-mito-TPP, that aggregate inside cancer cell mitochondria driven by higher negative membrane potential and undergoes aggregation-induced emission, later leading to apoptosis and (**B**) intramitochondrial biomineralization of silane containing compounds that polymerize inside the mitochondria driven by the higher accumulation and slightly basic pH (~8) of the mitochondrial matrix. (Copyright permission received from [69] and we thank RSC for figure 6B from [70]).

An aggregating molecule with aggregation-induced emission (AIE) property in mitochondria was introduced by Liu et al. The higher negative membrane potential of cancer mitochondria (about 60 mV over their normal counterparts) increases the accumulation of the designed molecule AIE-mito-TPP in cancer mitochondria compared with normal mitochondria, providing high selectivity (Figure 6A). This molecule serves as a light-up probe for image-guided therapy by conjugating TPP with a fluorogen, which can undergo AIE. The probe AIE-mito-TPP enters the target mitochondria and self-aggregates to emit fluorescence. AIE-mito-TPP induces cancer cell death as a result of membrane damage, ROS generation, and ATP depletion. In contrast, normal cells remain unaffected under similar treatment conditions [69]. Meanwhile, Kim et al. developed a biomineralization approach inside mitochondria for inducing apoptosis of cancer cells. Biomineralization, the process by which a living organism produces minerals, is an essential process for the generation of bio objects such as bones and teeth. Cellular functions are also regulated by isolation and secretion of intracellular mineral deposits. Although the potential use of extracellular biomineralization was recently reported, abnormal mineral deposits could inflict damage to normal cells near the nucleation sites. To overcome this drawback, the combination of an intracellular targeting strategy with biomineralization could offer new theranostics with reduced side effects. A mitochondria-targeting biomineralization system was introduced by conjugating TPP into trialkoxysilane. The trialkoxysilane group undergoes an environment-responsive silicification process inside the slightly basic mitochondrial matrix (~pH 8) (Figure 6B). This biomineralization exhibits concentration-dependent property. In cancer cells, the building blocks can accumulate into mitochondria above critical concentration, but not in normal cells because of the TPP-driven targeting ability. The accumulated condition and mitochondrial basic environment provide the appropriate condition that favors biomineralization inside cancer mitochondria. The resulted biominerals induce mitochondrial stress, leading to dysfunction of mitochondria and activation of apoptotic pathways. The cell viability analysis of the mitochondria-targeted biomineralizing compound showed an IC_50_ of 10–20 μM in varies cancer cell lines, such as HeLa, MDA-MB-231, SCC7, PC3, and KB, which contrasts with the low toxicity exhibited by normal cells [70]. Both approaches showed less damage to normal cells, which is a major advantage of targeting cancer mitochondria by the drug-free approach.

### 3.2. In Situ Nanostructures Inside Mitochondria for Cancer Therapy

Mitochondria-targeted self-assembled peptides were developed by Wang et al. for multitargeting, high selectivity, and minimal drug resistance. The same group established enzyme-instructed self-assembly (EISA) as an alternative strategy for cancer therapy, which is a pioneering example of a drug-free approach for cancer therapy. In this approach, a peptide bearing enzyme-sensitive hydrophobic building unit transforms into a hydrophobic self-assembling unit via an enzyme–substrate reaction. The highly hydrophobic self-assembling peptides undergo self-assembly in the pericellular space, cell membrane, or inside the cell, leading to apoptosis [71]. To achieve mitochondria-targeted EISA for cancer therapy, the self-assembling peptide NBD-FFYpK-TPP was synthesized bearing 4-nitro-2,1,3-benzoxadiazole (NBD) as a fluorophore, FFYK (phe-phe-tyr-lys)as a self-assembling peptide unit, tyrosine phosphate (Yp) as an ALP (alkaline phosphatase) enzymatic substrate, and TPP as a mitochondria-targeting unit. The hydrophilic peptide turns to hydrophobic with high self-assembling propensity near the cell membrane due to the dephosphorylation of Yp by the action of ALP, which is expressed in the cell membrane. The peptide self-assembles into nanofibers and is internalized into target mitochondria via endocytosis. The nanofibers induce cell death by initiating an intrinsic apoptotic cascade [72]. The repeated stimulation of ALP overexpressed cells by NBD-FFYpK-TPP showed similar cytotoxicity to that of the unstimulated cells, suggesting the lack of acquired resistance to the cells.

**Figure 7:**
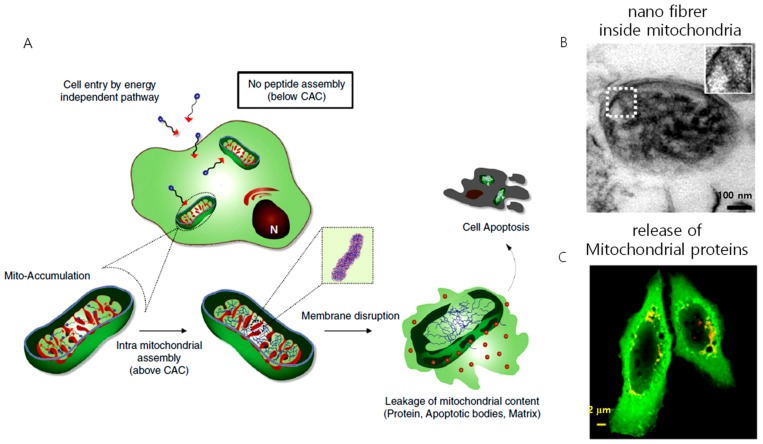
(**A**) Schematic illustration of mitochondria localization induced self-assembly of peptide amphiphiles. The peptides accumulate inside the mitochondria, exceed the critical aggregation concentration (CAC), and result in the formation of nanofibers. The nanofiber interacts with the mitochondrial membrane, induces the release of proteins, apoptotic factors leading to apoptosis of the cell, (**B**) TEM image of nanofiber inside the mitochondria, (**C**) release of proteins from the mitochondria to the cytosol after treating with an intra-mitochondrially self-assembling peptide.(Copyright permission received from [73]).

One concern about pericellular self-assembly is that the peptide requires a high concentration in the range of several hundred micromolar (μM) to millimolar (mM) to self-assemble inside the cell or pericellular space, and the enzymatic expression varies from cell to cell, which hampers its application as a therapeutic agent. This issue was addressed by Jeena et al. by introducing mitochondria localization-induced self-assembly for cancer therapy (Figure 7). In their work, the targeted amphiphilic peptide self-assembles inside the mitochondria to form nanofibers. The intramitochondrial self-assembly of the designed peptide was induced by the high local concentration inside the confined space. The size of mitochondria is very small, ranging from 0.5 µm to 1 µm, compared to the size of the whole cell (~15–20 µm). As a result, inside the mitochondria, the peptide experiences a 100–500-fold increment in concentration. The Py-FFK-TPP (Mito-FF) peptide was synthesized as a model peptide. Pyrene butyric acid (Py) was conjugated for the fluorescent detection inside the cell and TPP for mitochondria targeting. The peptide formed fibers of 10 nm in diameter under physiological conditions. A cellular incubation showed that the peptide localized inside the mitochondria, raising its concentration to 2.6 mM for an incubation concentration of 10 µM, which induced rapid self-assembly into nanofibers inside the mitochondria. The self-assembled fibers disrupted the mitochondria membrane to induce apoptosis. This approach constitutes an alternative strategy to overcome challenges imposed on cancer therapy, such as drug resistance and solubility issues [73]. Furthermore, the coincubation of both L and D isomers, i.e., Mito-FF and Mito-ff in 1:1 ratio (racemic mixture, Mito-rac) induced the formation of superstructures inside the mitochondria as a result of their heterochiral coassembly [74]. The superstructured fibrils induced selective damage of cancer mitochondria much more efficiently than the enantiomeric analog both in vitro and in vivo, resulting in enhanced cytotoxicity and tumor inhibition capability. The enhanced cellular damage of Mito-rac is caused by the higher area and depth of mitochondrial membrane penetration by the superstructured fibrils of 30–100 nm in diameter and several micrometers in length. Several reports describe that the heterochiral self-assembly of peptides produces nanostructures with enhanced mechanical property and enzymatic stability. However, this is the first report showing heterochiral self-assembly in the cellular environment for therapeutics and offers a new approach for tuning the biological activity of peptides by combining D and L isomers in different ratios. In both systems, cell death was induced by destroying mitochondrial membrane integrity. The fibers formed in situ interact with the mitochondrial membrane and induce the formation of pores by vertically penetrating the mitochondrial membrane, causing the release of apoptotic bodies that leads to cell death.

Small molecule-based anticancer agents are usually rapidly cleared from the body, reducing targeting efficiency. However, nano assemblies, such as nanoparticle-based therapy, offer high circulation and tumor targeting by the EPR effect. Wang et al. constructed a polymer–peptide conjugate consisting of a β-sheet-forming peptide (KLVFF), a mitochondria-targeting peptide (KLAK), and polyvinyl alcohol (PVA) as a backbone. The designed nanomaterial forms nanoparticles under physiological conditions that are responsive toward ROS, transforming into nanofibers near the mitochondria. This morphology transformation is triggered by a change in the hydrophobic–hydrophilic balance. The polymer–peptide complex showed high mitochondrial disruption ability against HeLa cells, with an IC_50_ value of about 10 μg/mL, much lower than that of free KLAK and other controls. The morphology transformation is operative under in vivo conditions. In the in vivo system, a Cy5-labeled complex was shown to accumulate at the tumor site and reach the highest intensity in 24 h [75]. He et al. reported a branched peptide targeting mitochondria that carries a negative charge. A self-assembling motif conjugated with a protein tag (FLAG-tag) forms micelles. The protein tag FLAG (DDDDK) is cleaved by the enzyme entarokinase (ENTK), which transforms micelle to nanofibers. This transformation occurs in the intracellular environment, and the as-formed nanofibers are located in the mitochondria. The FLAG-tag precursor delivers cargo to the mitochondria, as evidenced by the delivery of DOX or RPE (R-phycoerythrin). The branched peptide precursor decreases the IC_50_ of DOX from 3.0 to 400 nM [76].

## 4. Conclusions and Future Outlook

Hundreds of strategies to achieve cancer-selective mitochondrial damage leading to apoptosis have been introduced so far. The conventional strategies of targeting mitochondria for cancer therapy include mitochondria-targeted cytotoxic drugs, mitochondrial protein inhibitors, and mitochondria-localized PDT, offering excellent cancer mitochondria-targeting/damaging efficiency. The modern strategy of targeting mitochondria in drug-free approaches promises advantages, such as lack of acquired resistance, higher selectivity, and lower working concentration. Generally, drug-free approaches do not rely on a specific protein target inside mitochondria but damage its structural or functional features, which render such approaches less prone to acquire drug resistance. Table 3 summarizes the barriers and advantages of different mitochondria-targeted drugs towards the clinical trial.

However, certain challenges still need to be overcome for the ideal performance of the drug-free strategy. First, for a molecule to penetrate the mitochondrial membrane, it requires positive charge and lipophilicity, which often results in molecular uptake by normal cells as well. Second, the presence of a positive charge in the mitochondria-targeting compounds limits their applicability under in vivo conditions, since the negatively charged enzymes or proteins could interact with mitochondriotropic agents, reducing the targeting efficiency. To improve the performance of the drug-free approaches, the mitochondria localization-induced self-assembly of the amphiphilic peptide can be envisaged as a powerful strategy; however, drawbacks such as the fast clearance of small molecules from the body during in vivo administration, poor selectivity, and nonspecific interaction of positive charges hinder its applicability. To overcome this issue, the mitochondria localization-induced self-assembly could be integrated with stimuli responsiveness, such as enzyme or pH for enhancing the targeting efficiency, and the construction of nanostructures with intra-mitochondrially assembling molecules could circumvent the problems of blood clearance and off-target effect. This combination of stimuli responsiveness or nanostructure with the desired mitochondria-targeted drug-free strategy greatly enhances the performance. In the future, further research on the design and development of mitochondria-targeted drug-free compounds or strategies is highly expected to result in clinical trials for efficient and side-effect-free cancer therapeutics.

## Figures and Tables

**Table 1:** Summary of compounds that target mitochondrial metabolism/proteins leading to apoptosis.

No.	Mitochondrial Protein/Metabolism Targeted Drug	Target of Interest	Mode of Cell Death	Reference
1	alpha-tocopheryl succinate (α-TOS)	inhibits succinate dehydrogenase (SDH)	ROS accumulation apoptosis.	[36]
2	resveratrol	inhibits complex III, induce the expression of mitochondrial superoxide dismutase (SOD2)	apoptosis	[38]
3	clodronate	inhibit the activity of adenine nucleotide transporter (ANT), inhibit mitochondrial oxygen consumption	apoptosis	[40]
4	AppCCl_2_P	ADP/ATP translocation	apoptosis	[40]
5	ABT-737	inhibits anti-apoptotic proteins, disturb mitochondrial membrane potential, increase intra cellular ROS	apoptosis	[43]
6	SMTIN-P01	inhibits mitochondrial TRAP1	apoptosis	[44]
7	TPP conjugated terpyridine-Pt	inhibits thioredoxin (TrxR)	apoptosis	[45]

**Table 2:** Advantages and disadvantages of different mitochondria-targeted conventional approaches.

No.	Molecular Design	Advantages	Disadvantages
1	Conventional drug conjugate with mitochondria targeting ligand	Enhanced cell cytotoxicity	Induces toxicity towards normal cell as well
2	Photosensitizer conjugate with mitochondria targeting ligand	Quick action, higher toxicity	Induces toxicity towards normal cell as well, requires an external aid of laser for PS activation
3	Protein inhibitor conjugate with mitochondria targeting ligand	High cancer selectivity	Induces acquired resistance as a result of targeted protein mutation upon repeated administration
4	Mitochondria penetrating peptide conjugate with cargo	High mitochondria penetrating ability	Could penetrate normal cells as well, cannot be used for higher molecular weight cargo, complicated design and synthesis.

**Table 3:** Analysis of the possibility of different mitochondria-targeted molecules to be available for the practical application.

No.	Molecular Design	Barrier for Practical Application	Advantages for Practical Application
1	Conventional drug conjugate with mitochondria targeting ligand	Fetal toxicity and side effects	-
2	Protein inhibitor conjugate with mitochondria targeting ligand	Complicated synthetic procedure, acquired resistance	High tumor selectivity
3	Mitochondria penetrating peptide conjugate with cargo	Complicated molecular design, needs to carry a cargo.	-
4	Mitochondria-targeted drug-free agents	-	Work at small concertation, ease of production, high selectivity towards the tumor, fewer side effects.

## References

[1] Hoye A.T., Davoren J.E., Wipf P., Fink M.P., Kagan V.E. (2008). Taegeting Mitochondria. Acc. Chem. Res..

[2] Golstein P., Kroemer G. (2007). Cell death by necrosis: Towards a molecular definition. Trends Biochem. Sci..

[3] Weinberg S.E., Chandel N.S. (2015). Targeting mitochondria metabolism for cancer therapy. Nat. Chem. Biol..

[4] Yousif L.F., Stewart K.M., Kelley S.O. (2009). Targeting mitochondria with organelle-specific compounds: Strategies and applications. Chembiochem.

[5] Porporato P.E., Filigheddu N., Pedro J.M.B., Kroemer G., Galluzzi L. (2018). Mitochondrial metabolism and cancer. Cell Res..

[6] Chen J., Jiang X., Zhang C., MacKenzie K.R., Stossi F., Palzkill T., Wang M.C., Wang J. (2017). Reversible Reaction-Based Fluorescent Probe for Real-Time Imaging of Glutathione Dynamics in Mitochondria. ACS Sens..

[7] Kroemer G. (2003). Mitochondrial control of apoptosis: An introduction. Biochem. Biophys. Res. Commun..

[8] Cai J., Yang J., Jones D.P. (1998). Mitochondrial control of apoptosis: The role of cytochrome C. Biochim. Biophys. Acta.

[9] Tsujimoto Y., Nakagawa T., Shimizu S. (2006). Mitochondrial membrane permeability transition and cell death. Biochim. Biophys. Acta.

[10] Baines C.P. (2010). Role of the mitochondrion in programmed necrosis. Front. Physiol..

[11] Giorgi C., Agnoletto C., Bononi A., Bonora M., De Marchi E., Marchi S., Missiroli S., Patergnani S., Poletti F., Rimessi A. (2012). Mitochondrial calcium homeostasis as potential target for mitochondrial medicine. Mitochondrion.

[12] Kalkavan H., Green D.R. (2018). MOMP, cell suicide as a BCL-2 family business. Cell Death Differ..

[13] Tait S.W., Green D.R. (2013). Mitochondrial regulation of cell death. Cold Spring Harb. Perspect. Biol..

[14] Kroemer G. (2006). Mitochondria in cancer. Oncogene.

[15] Wisnovsky S., Lei E.K., Jean S.R., Kelley S.O. (2016). Mitochondrial Chemical Biology: New Probes Elucidate the Secrets of the Powerhouse of the Cell. Cell Chem. Biol..

[16] Parker S.J., Metallo C.M. (2015). Metabolic consequences of oncogenic IDH mutations. Pharmacol. Ther..

[17] Wallace D.C. (2012). Mitochondria and cancer. Nat. Rev. Cancer.

[18] Rin Jean S., Tulumello D.V., Wisnovsky S.P., Lei E.K., Pereira M.P., Kelley S.O. (2014). Molecular vehicles for mitochondrial chemical biology and drug delivery. ACS Chem. Biol..

[19] Teixeira J., Oliveira C., Cagide F., Amorim R., Garrido J., Borges F., Oliveira P.J. (2018). Discovery of a new mitochondria permeability transition pore (mPTP) inhibitor based on gallic acid. J. Enzym. Inhib. Med. Chem..

[20] Dilip A., Cheng G., Joseph J., Kunnimalaiyaan S., Kalyanaraman B., Kunnimalaiyaan M., Gamblin T.C. (2013). Mitochondria-targeted antioxidant and glycolysis inhibition: Synergistic therapy in hepatocellular carcinoma. Anticancer Drugs.

[21] Mannella C.A. (2006). Structure and dynamics of the mitochondrial inner membrane cristae. Biochim. Biophys. Acta.

[22] Millard M., Pathania D., Shabaik Y., Taheri L., Deng J., Neamati N. (2010). Preclinical evaluation of novel triphenylphosphonium salts with broad-spectrum activity. PLoS ONE.

[23] Zorova L.D., Popkov V.A., Plotnikov E.Y., Silachev D.N., Pevzner I.B., Jankauskas S.S., Babenko V.A., Zorov S.D., Balakireva A.V., Juhaszova M. (2018). Mitochondrial membrane potential. Anal. Biochem..

[24] Zielonka J., Joseph J., Sikora A., Hardy M., Ouari O., Vasquez-Vivar J., Cheng G., Lopez M., Kalyanaraman B. (2017). Mitochondria-Targeted Triphenylphosphonium-Based Compounds: Syntheses, Mechanisms of Action, and Therapeutic and Diagnostic Applications. Chem. Rev..

[25] Liu D., Jin F., Shu G., Xu X., Qi J., Kang X., Yu H., Lu K., Jiang S., Han F. (2019). Enhanced efficiency of mitochondria-targeted peptide SS-31 for acute kidney injury by pH-responsive and AKI-kidney targeted nanopolyplexes. Biomaterials.

[26] Sweetwyne M.T., Pippin J.W., Eng D.G., Hudkins K.L., Chiao Y.A., Campbell M.D., Marcinek D.J., Alpers C.E., Szeto H.H., Rabinovitch P.S. (2017). The mitochondrial-targeted peptide, SS-31, improves glomerular architecture in mice of advanced age. Kidney Int..

[27] Horton K.L., Stewart K.M., Fonseca S.B., Guo Q., Kelley S.O. (2008). Mitochondria-penetrating peptides. Chem. Biol..

[28] Lincoln R., Greene L.E., Zhang W., Louisia S., Cosa G. (2017). Mitochondria Alkylation and Cellular Trafficking Mapped with a Lipophilic BODIPY-Acrolein Fluorogenic Probe. J. Am. Chem. Soc..

[29] Liu Y., Zhou J., Wang L., Hu X., Liu X., Liu M., Cao Z., Shangguan D., Tan W. (2016). A Cyanine Dye to Probe Mitophagy: Simultaneous Detection of Mitochondria and Autolysosomes in Live Cells. J. Am. Chem. Soc..

[30] Biswas S., Dodwadkar N.S., Deshpande P.P., Torchilin V.P. (2012). Liposomes loaded with paclitaxel and modified with novel triphenylphosphonium-PEG-PE conjugate possess low toxicity, target mitochondria and demonstrate enhanced antitumor effects in vitro and in vivo. J. Control. Release.

[31] Han X., Su R., Huang X., Wang Y., Kuang X., Zhou S., Liu H. (2019). Triphenylphosphonium-modified mitochondria-targeted paclitaxel nanocrystals for overcoming multidrug resistance. Asian J. Pharm. Sci..

[32] Han M., Vakili M.R., Soleymani Abyaneh H., Molavi O., Lai R., Lavasanifar A. (2014). Mitochondrial delivery of doxorubicin via triphenylphosphine modification for overcoming drug resistance in MDA-MB-435/DOX cells. Mol. Pharm..

[33] Jin L., Dai L., Ji M., Wang H. (2019). Mitochondria-targeted triphenylphosphonium conjugated glycyrrhetinic acid derivatives as potent anticancer drugs. Bioorg. Chem..

[34] Peng Y.B., Zhao Z.L., Liu T., Xie G.J., Jin C., Deng T.G., Sun Y., Li X., Hu X.X., Zhang X.B. (2017). A Multi-Mitochondrial Anticancer Agent that Selectively Kills Cancer Cells and Overcomes Drug Resistance. ChemMedChem.

[35] Reshetnikov V., Daum S., Janko C., Karawacka W., Tietze R., Alexiou C., Paryzhak S., Dumych T., Bilyy R., Tripal P. (2018). ROS-Responsive N-Alkylaminoferrocenes for Cancer-Cell-Specific Targeting of Mitochondria. Angew. Chem..

[36] Stapelberg M., Gellert N., Swettenham E., Tomasetti M., Witting P.K., Procopio A., Neuzil J. (2005). Alpha-tocopheryl succinate inhibits malignant mesothelioma by disrupting the fibroblast growth factor autocrine loop: Mechanism and the role of oxidative stress. J. Biol. Chem..

[37] Dong L.F., Low P., Dyason J.C., Wang X.F., Prochazka L., Witting P.K., Freeman R., Swettenham E., Valis K., Liu J. (2008). Alpha-tocopheryl succinate induces apoptosis by targeting ubiquinone-binding sites in mitochondrial respiratory complex II. Oncogene.

[38] Juan M.E., Wenzel U., Daniel H., Planas J.M. (2008). Resveratrol induces apoptosis through ROS-dependent mitochondria pathway in HT-29 human colorectal carcinoma cells. J. Agric. Food Chem..

[39] Chipuk J.E., Bouchier-Hayes L., Green D.R. (2006). Mitochondrial outer membrane permeabilization during apoptosis: The innocent bystander scenario. Cell Death Differ..

[40] Modica-Napolitano J.S., Singh K.K. (2002). Mitochondria as targets for detection and treatment of cancer. Expert Rev. Mol. Med..

[41] Lehenkari P.P., Kellinsalmi M., Napankangas J.P., Ylitalo K.V., Monkkonen J., Rogers M.J., Azhayev A., Vaananen H.K., Hassinen I.E. (2002). Further insight into mechanism of clodronate: Inhibition of mitochondrial ADP/ATP translocase by a nonhydrolyzable, adnine-containing metabolite. Mol. Pharmacol..

[42] Kang M.H., Reynolds C.P. (2009). Bcl-2 inhibitors: Targeting mitochondrial apoptotic pathways in cancer therapy. Clin. Cancer Res..

[43] Oltersdorf T., Elmore S.W., Shoemaker A.R., Armstrong R.C., Augeri D.J., Belli B.A., Bruncko M., Deckwerth T.L., Dinges J., Hajduk P.J. (2005). An inhibitor of Bcl-2 family proteins induces regression of solid tumours. Nature.

[44] Lee C., Park H.K., Jeong H., Lim J., Lee A.J., Cheon K.Y., Kim C.S., Thomas A.P., Bae B., Kim N.D. (2015). Development of a mitochondria-targeted Hsp90 inhibitor based on the crystal structures of human TRAP1. J. Am. Chem. Soc..

[45] Wang K., Zhu C., He Y., Zhang Z., Zhou W., Muhammad N., Guo Y., Wang X., Guo Z. (2019). Restraining Cancer Cells by Dual Metabolic Inhibition with a Mitochondrion-Targeted Platinum(II) Complex. Angew. Chem..

[46] Mandal D., Nasrolahi Shirazi A., Parang K. (2014). Self-assembly of peptides to nanostructures. Org. Biomol. Chem..

[47] Zhao X., Pan F., Xu H., Yaseen M., Shan H., Hauser C.A., Zhang S., Lu J.R. (2010). Molecular self-assembly and applications of designer peptide amphiphiles. Chem. Soc. Rev..

[48] Lowik D.W., van Hest J.C. (2004). Peptide based amphiphiles. Chem. Soc. Rev..

[49] Lei E.K., Kelley S.O. (2017). Delivery and Release of Small-Molecule Probes in Mitochondria Using Traceless Linkers. J. Am. Chem. Soc..

[50] Chamberlain G.R., Tulumello D.V., Kelley S.O. (2013). Targeted delivery of doxorubicin to mitochondria. ACS Chem. Biol..

[51] Jean S.R., Tulumello D.V., Riganti C., Liyanage S.U., Schimmer A.D., Kelley S.O. (2015). Mitochondrial Targeting of Doxorubicin Eliminates Nuclear Effects Associated with Cardiotoxicity. ACS Chem. Biol..

[52] Wisnovsky S.P., Wilson J.J., Radford R.J., Pereira M.P., Chan M.R., Laposa R.R., Lippard S.J., Kelley S.O. (2013). Targeting mitochondrial DNA with a platinum-based anticancer agent. Chem. Biol..

[53] Yang C., Hu R., Lu F., Guo X., Wang S., Zeng Y., Li Y., Yang G. (2019). Traceable cancer cell photoablation with a new mitochondria-responsive and -activatable red-emissive photosensitizer. Chem. Commun..

[54] Hilf R. (2007). Mitochondria are targets of photodynamic therapy. J. Bioenerg. Biomembr..

[55] Yang L., Gao P., Huang Y., Lu X., Chang Q., Pan W., Li N., Tang B. (2019). Boosting the photodynamic therapy efficiency with a mitochondria-targeted nanophotosensitizer. Chin. Chem. Lett..

[56] Ethirajan M., Chen Y., Joshi P., Pandey R.K. (2011). The role of porphyrin chemistry in tumor imaging and photodynamic therapy. Chem. Soc. Rev..

[57] Xia D., Xu P., Luo X., Zhu J., Gu H., Huo D., Hu Y. (2019). Overcoming Hypoxia by Multistage Nanoparticle Delivery System to Inhibit Mitochondrial Respiration for Photodynamic Therapy. Adv. Funct. Mater..

[58] Lv W., Zhang Z., Zhang K.Y., Yang H., Liu S., Xu A., Guo S., Zhao Q., Huang W. (2016). A Mitochondria-Targeted Photosensitizer Showing Improved Photodynamic Therapy Effects Under Hypoxia. Angew. Chem..

[59] Yi S., Lu Z., Zhang J., Wang J., Xie Z., Hou L. (2019). Amphiphilic Gemini Iridium(III) Complex as a Mitochondria-Targeted Theranostic Agent for Tumor Imaging and Photodynamic Therapy. ACS Appl. Mater. Interfaces.

[60] Zhou Z., Liu J., Rees T.W., Wang H., Li X., Chao H., Stang P.J. (2018). Heterometallic Ru-Pt metallacycle for two-photon photodynamic therapy. Proc. Natl. Acad. Sci. USA.

[61] Tian N., Sun W., Guo X., Lu J., Li C., Hou Y., Wang X., Zhou Q. (2019). Mitochondria targeted and NADH triggered photodynamic activity of chloromethyl modified Ru(ii) complexes under hypoxic conditions. Chem. Commun..

[62] Chakrabortty S., Agrawalla B.K., Stumper A., Vegi N.M., Fischer S., Reichardt C., Kogler M., Dietzek B., Feuring-Buske M., Buske C. (2017). Mitochondria Targeted Protein-Ruthenium Photosensitizer for Efficient Photodynamic Applications. J. Am. Chem. Soc..

[63] Li M., Li X., Cao Z., Wu Y., Chen J.A., Gao J., Wang Z., Guo W., Gu X. (2018). Mitochondria-targeting BODIPY-loaded micelles as novel class of photosensitizer for photodynamic therapy. Eur. J. Med. Chem..

[64] Noh I., Lee D., Kim H., Jeong C.U., Lee Y., Ahn J.O., Hyun H., Park J.H., Kim Y.C. (2018). Enhanced Photodynamic Cancer Treatment by Mitochondria-Targeting and Brominated Near-Infrared Fluorophores. Adv. Sci..

[65] Thomas A.P., Palanikumar L., Jeena M.T., Kim K., Ryu J.H. (2017). Cancer-mitochondria-targeted photodynamic therapy with supramolecular assembly of HA and a water soluble NIR cyanine dye. Chem. Sci..

[66] Feng R.-M., Zong Y.-N., Cao S.-M. (2019). Current cancer situation in China: Good or bad news from the 2018 global cancer statics?. Cancer Commun..

[67] Housman G., Byler S., Heerboth S., Lapinska K., Longacre M., Snyder N., Sarkar S. (2014). Drug resisitance in cancer: An overview. Cancers.

[68] Zhao R., Wang B., Yang X., Xiao Y., Wang X., Shao C., Tang R. (2016). A drug-free tumor therapy strategy: Cancer-cell-targeting calcification. Angew. Chem..

[69] Hu Q., Gao M., Feng G., Liu B. (2014). Mitochondria-targeted cancer therapy using a light-up probe with aggregation-induced-emission characteristics. Angew. Chem..

[70] Kim S., Palanikumar L., Choi H., Jeena M.T., Kim C., Ryu J.H. (2018). Intra-mitochondrial biomineralization for inducing apoptosis of cancer cells. Chem. Sci..

[71] Kuang Y., Shi J., Li J., Yuan D., Alberti K.A., Xu Q., Xu B. (2014). Pericellular hydrogel/nanonets inhibit cancer cells. Angew. Chem..

[72] Wang H., Feng Z., Wang Y., Zhou R., Yang Z., Xu B. (2016). Integrating Enzymatic Self-Assembly and Mitochondria Targeting for Selectively Killing Cancer Cells without Acquired Drug Resistance. J. Am. Chem. Soc..

[73] Jeena M.T., Palanikumar L., Go E.M., Kim I., Kang M.G., Lee S., Park S., Choi H., Kim C., Jin S.M. (2017). Mitochondria localization induced self-assembly of peptide amphiphiles for cellular dysfunction. Nat. Commun..

[74] Jeena M.T., Jeong K., Go E.M., Cho Y., Lee S., Jin S., Hwang S.-K., Jang J.H., Kang C.S., Bang W.-Y. (2019). Heterochiral Assembly of Amphiphilic Peptides Inside the Mitochondria for Supramolecular Cancer Therapeutics. ACS Nano.

[75] Cheng D.B., Zhang X.H., Gao Y.J., Ji L., Hou D., Wang Z., Xu W., Qiao Z.Y., Wang H. (2019). Endogenous Reactive Oxygen Species-Triggered Morphology Transformation for Enhanced Cooperative Interaction with Mitochondria. J. Am. Chem. Soc..

[76] He H., Wang J., Wang H., Zhou N., Yang D., Green D.R., Xu B. (2018). Enzymatic Cleavage of Branched Peptides for Targeting Mitochondria. J. Am. Chem. Soc..

